# The association of polygenic risk for schizophrenia, bipolar disorder, and depression with neural connectivity in adolescents and young adults: examining developmental and sex differences

**DOI:** 10.1038/s41398-020-01185-7

**Published:** 2021-01-14

**Authors:** J. L. Meyers, D. B. Chorlian, T. B. Bigdeli, E. C. Johnson, F. Aliev, A. Agrawal, L. Almasy, A. Anokhin, H. J. Edenberg, T. Foroud, A. Goate, C. Kamarajan, S. Kinreich, J. Nurnberger, A. K. Pandey, G. Pandey, M. H. Plawecki, J. E. Salvatore, J. Zhang, A. Fanous, B. Porjesz

**Affiliations:** 1 Department of Psychiatry, State University of New York (SUNY) Downstate Health Sciences University, Brooklyn, NY 11203 USA; 2 Department of Psychiatry, Washington University School of Medicine, St. Louis, MO 63110 USA; 3 Department of Psychology & College Behavioral and Emotional Health Institute, Virginia Commonwealth University, Richmond, VA 23284 USA; 4 Faculty of Business, Karabuk University, Karabuk, Turkey; 5 Department of Genetics, Perelman School of Medicine, University of Pennsylvania, Philadelphia, PA 19104 USA; 6 Department of Medical and Molecular Genetics, Indiana University School of Medicine, Indianapolis, IN 46202 USA; 7 Department of Biochemistry and Molecular Biology, Indiana University School of Medicine, Indianapolis, IN 46202 USA; 8 Departments of Neuroscience, Icahn School of Medicine at Mount Sinai, New York, NY 10029 USA; 9 Departments of Genetics and Genomic Sciences, Icahn School of Medicine at Mount Sinai, New York, NY 10029 USA; 10 Virginia Institute of Psychiatric and Behavioral Genetics, Department of Psychiatry, Virginia Commonwealth University, Richmond, VA 23284 USA

**Keywords:** Genomics, Schizophrenia

## Abstract

Neurodevelopmental abnormalities in neural connectivity have been long implicated in the etiology of schizophrenia (SCZ); however, it remains unclear whether these neural connectivity patterns are associated with genetic risk for SCZ in unaffected individuals (i.e., an absence of clinical features of SCZ or a family history of SCZ). We examine whether polygenic risk scores (PRS) for SCZ are associated with functional neural connectivity in adolescents and young adults without SCZ, whether this association is moderated by sex and age, and if similar associations are observed for genetically related neuropsychiatric PRS. One-thousand four-hundred twenty-six offspring from 913 families, unaffected with SCZ, were drawn from the Collaborative Study of the Genetics of Alcoholism (COGA) prospective cohort (median age at first interview = 15.6 (12–26), 51.6% female, 98.1% European American, 41% with a family history of alcohol dependence). Participants were followed longitudinally with resting-state EEG connectivity (i.e., coherence) assessed every two years. Higher SCZ PRS were associated with elevated theta (3–7 Hz) and alpha (7–12 Hz) EEG coherence. Associations differed by sex and age; the most robust associations were observed between PRS and parietal-occipital, central-parietal, and frontal-parietal alpha coherence among males between ages 15–19 (*B*: 0.15–0.21, *p* < 10^–4^). Significant associations among EEG coherence and Bipolar and Depression PRS were observed, but differed from SCZ PRS in terms of sex, age, and topography. Findings reveal that polygenic risk for SCZ is robustly associated with increased functional neural connectivity among young adults without a SCZ diagnosis. Striking differences were observed between men and women throughout development, mapping onto key periods of risk for the onset of psychotic illness and underlining the critical importance of examining sex differences in associations with neuropsychiatric PRS across development.

## Introduction

Schizophrenia (SCZ) is a debilitating neuropsychiatric syndrome with typical onset during the second decade of life, and characterized by pronounced deficits in sensory and cognitive processing^1^. Hypotheses regarding its neurodevelopmental origins date back to Kraepelin, and later studies of high-risk children describe neurological “dysmaturation” during early childhood^2^. Genetic risk for SCZ, as measured by polygenic risk scores (PRS), and brain functional connectivity have been studied among those affected with SCZ^3–5^. However, investigating neurodevelopmental patterns of connectivity in *unaffected* adolescents and young adults, at varying levels of polygenic susceptibility, may uncover basic patterns of brain connectivity that could underlie development of the disorder. This information can elucidate basic neurobiological mechanisms by which the variants that contribute to genetic risk for SCZ affect brain connectivity and development patterns.

Disordered cortical connectivity is now considered a central feature of SCZ, with evidence from human and animal studies demonstrating structural and functional *dysconnectivity* between several brain regions^6–8^. Neural functional connectivity measured using EEG has the advantage of temporal resolution on the order of milliseconds, the scale at which most relevant sensory, motor and cognitive phenomena occur^8^. EEG coherence measures functional connectivity by the degree of synchrony in oscillatory activity between two brain regions, with increased coherence indicating functional integration between these brain regions and decreased coherence reflecting less correlated neural activity^9,10^. Stated another way, EEG coherence reflects communication between distinct brain regions, which facilitate cognition and behavior^11–14^. Either extreme of this spectrum may indicate disturbances in neural interactions underlying cognition, particularly in the absence of task-related activity (e.g., Default Mode Network^15,16^). Differences in resting-state EEG coherence have been observed in individuals with SCZ and in their relatives^5^, with studies most consistently showing increases in intrahemispheric and interhemispheric coherence in the theta (3–7 Hz), alpha (7–12 Hz) frequencies^5^, among mixed evidence of mis-connectivity among affected individuals^7,17,18^. We note that differences in other frequency bands, including the gamma frequency (28–50 Hz) have been reported for spectral power and other aspects of EEG. These inconsistent results may result from small and heterogenous samples, since neural connectivity changes dynamically across the lifespan^19^, with relatively random network structures in childhood changing to more ordered networks in adolescence and emerging adulthood^20^. For these reasons, coherence has been particularly useful in the study of normal brain development and neuropsychiatric traits^17,21^, and in genomic studies of neurodevelopment and mental health conditions^22^. However, few studies have focused on neurodevelopmental trajectories among adolescents and young adults, some of whom may be prior to their onset of SCZ, but the vast majority of whom will remain unaffected. Such study designs allow a prospective view of future illness but are also relatively uncontaminated by neurobiological sequelae of the illness or its treatment.

The past decade has seen immense progress in psychiatric genetics, with genome-wide association studies (GWAS) having now identified 245 distinct risk loci for SCZ and the demonstration of polygenic influences on SCZ^3,4^. Polygenic risk scores (PRS) aggregate genetic information from large GWAS^3^, and index individuals’ polygenic liability for a given trait or disorder in an independent sample. Recent studies have shown that polygenic risk for SCZ influences aspects of executive functioning in individuals with^23^ and without SCZ^24^, albeit with limited effect sizes. By harnessing the polygenicity underlying SCZ and genetically correlated disorders (e.g., Bipolar Disorder (BiP) and Major Depressive Disorder (MDD)^25^), PRS can be leveraged to enhance our understanding of how genetic vulnerability to SCZ may manifest as individual differences in brain activity, providing critical interpretative context for emergent GWAS findings. Large GWAS have demonstrated, using PRS and genome-wide correlations, that there are considerable pleiotropic effects underlying SCZ, bipolar disorder (BiP), and major depressive disorder (MDD)^25^. By contrasting the SCZ PRS with PRS for genetically correlated disorders, such as BiP and MDD, the specificity and commonality of these genetic vulnerabilities on brain activity can be estimated. Although a previous study reported no association between SCZ PRS and classical EEG endophenotypes (e.g., P3 amplitude)^26^, no study has examined the influence of SCZ PRS on EEG connectivity in *unaffected* individuals. Further, no study has examined how these associations may differ by sex or across adolescence and young adulthood, the time-frame during which SCZ typically manifests. This is important, given research demonstrating sex and developmental differences in measures of EEG coherence^27,28^, the clinical presentation of SCZ^29^, and polygenic effects on neuropsychiatric illnesses^22,30,31^.

In the current study, we seek to uncover neurodevelopmental patterns of brain connectivity in *unaffected* (with SCZ, BiP, or MDD) adolescents and young adults as a function of their polygenic susceptibility for SCZ. We also aim to understand important sex and developmental differences in these brain connectivity patterns. We examined associations between polygenic risk for SCZ^32^ and neurodevelopmental trajectories of EEG coherence throughout adolescence and young adulthood among unaffected (nonpsychotic) individuals. Data was drawn from the Collaborative Study on the Genetics of Alcoholism (COGA), with longitudinal data available throughout a key period of risk for the onset of psychotic illness, ages 12–32. We also examined sex-specific effects and developmental differences in the association of SCZ PRS and EEG coherence that may be otherwise masked. Finally, we examined whether PRS for genetically correlated neuropsychiatric conditions, BiP and MDD, were differentially associated with neurodevelopmental trajectories of connectivity.

## Methods

### Sample and measures

COGA’s prospective study began data collection in 2004 and ended in 2019. Details of data collection and procedures have been published^33^. Briefly, offspring from families affected with AUD and community comparison families from the COGA study were enrolled when they were aged 12–22, with new subjects added as they reached the age of 12. Subjects were assessed ~every 2 years with a comprehensive battery that includes the Semi-Structured Assessment for the Genetics of Alcoholism (SSAGA)^34,35^ assessing substance use and related disorders, neurocognitive performance, and a neurophysiological battery that includes resting-state EEG. Owing to the majority European ancestry (EA) of the PGC SCZ GWAS, our analytic sample comprises EA individuals with genotypic and EEG data from at least three assessments (*N* = 1426 offspring from 913 EA families, with a mean of 3.5 assessments). Among them, the median age at first interview was 15.6 (Range = 12–26), 51.6% were female, 17.8% met criteria for DSM-IV Alcohol Dependence, and 41% had a family history of AUD. Experimental protocols were approved by each site’s IRB, and informed consent was obtained from all participants, including parental permission from participants aged 18 years or younger.

**Fig. 1:**
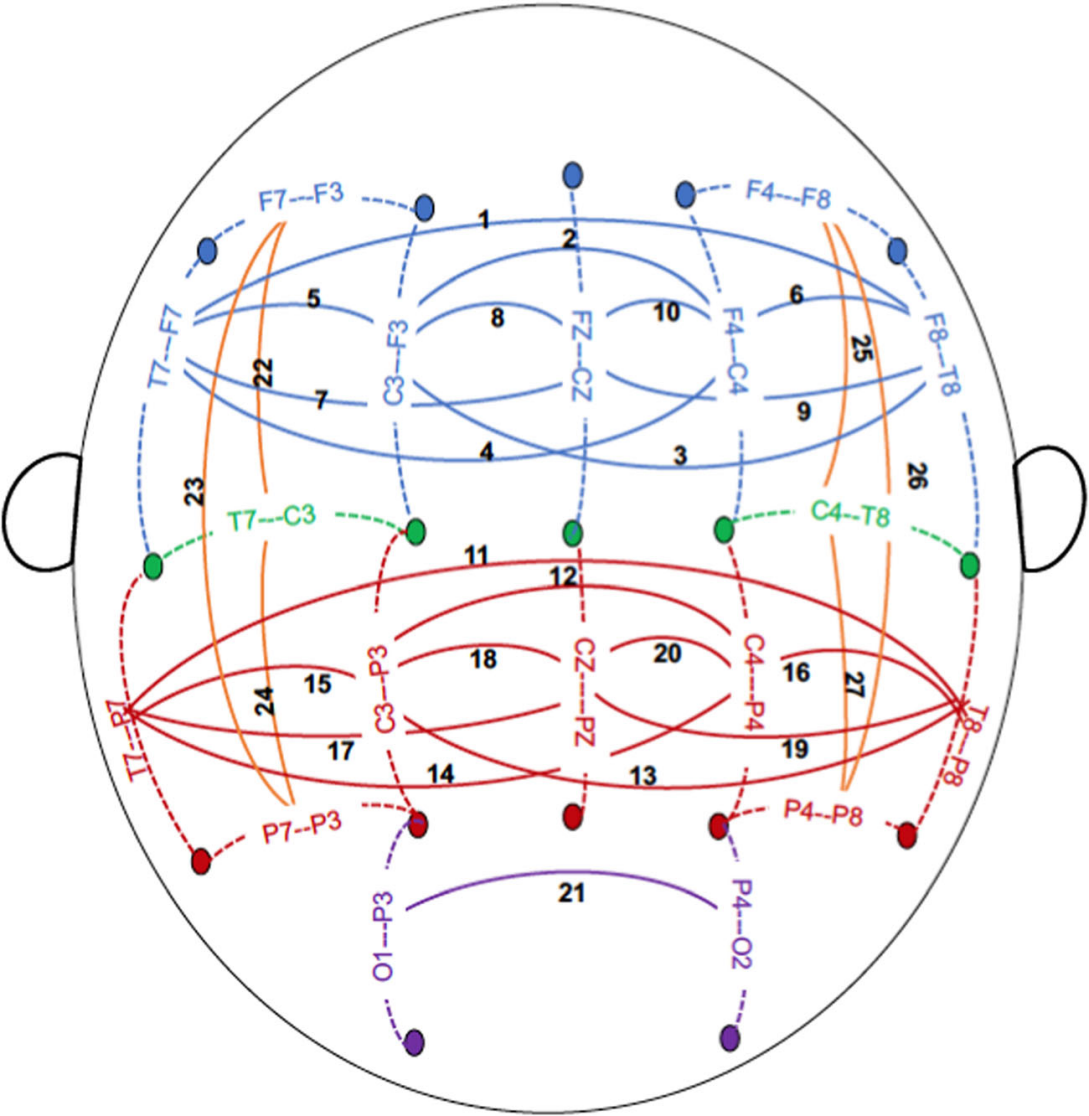
Scalp topography of EEG electrodes used for the computation of coherence. The schematic represents a top view of the scalp, nose up. Coherence (solid lines) was computed between bipolar EEG signals, each of which was derived from a pair of adjacent electrodes (connected by dotted lines). Electrode labeling: letters F, C, T, P, and O represent frontal, central, temporal, parietal, and occipital areas of the scalp, respectively; odd and even numbers represent the left and right hemispheres, respectively, and “Z” denotes electrodes along the sagittal midline. 27 Coherences between bipolar electrode pairs are illustrated as follows: frontal-central sagittal coherences are represented in blue; (1) F8-T8--F7-T7, (2) F4-C4--F3-C3, (3) F3-C3--F8-T8, (4) F4-C4--F7-T7, (5) F3-C3--F7-T7, (6) F4-C4--F8-T8, (7) FZ-CZ--F7-T7, (8) FZ-CZ--F3-C3, (9) FZ-CZ--F8-T8, (10) FZ-CZ--F4-C4. Central-parietal sagittal coherences are represented in red; (11) T8-P8--T7-P7, (12) C4-P4--C3-P3, (13) C3-P3--T8-P8, (14) C4-P4--T7-P7, (15) C3-P3--T7-P7, (16) C4-P4--T8-P8, (17) T7-P7--CZ-PZ, (18) C3-P3--CZ-PZ, (19) T8-P8--CZ-PZ, (20) C4-P4--CZ-PZ, parietal-occipital sagittal coherences are represented in purple; (21) P4-O2--P3-O1. Intrahemispheric lateral coherences are represented in orange; (22) T7-C3--F7-F3, (23) P7-P3--F7-F3, (24) P7-P3--T7-C3, (25) T8-C4--F8-F4, (26) P8-P4--F8-F4, (27) P8-P4--T8-C4.

EEG recording and processing have been detailed previously^36^. Briefly, resting (eyes-closed) EEG was recorded for 4.25 min; a continuous interval of 256 seconds was analyzed. Each subject wore a fitted electrode cap using the 64-channel montage as specified according to the 10–20 International system. The nose served as reference and the ground electrode was placed on the forehead. Electrode impedances were always maintained below 5 KOhm. EEG was recorded with subjects seated comfortably in a dimly lit sound-attenuated temperature-regulated booth. They were instructed to keep their eyes closed and remain relaxed, but not to fall asleep. Electrical activity was amplified 10,000 times by Neuroscan amplifiers, with a bandpass between 0.02 Hz and 100 Hz and recorded using the Neuroscan system (Compumedics Limited; El Paso, TX). EEG procedures were identical at all collection sites. Bipolar electrode pairs were derived to reduce volume conduction effects, and 27 representative coherence pairs (depicted in Fig. 1) were selected based on previous EEG coherence work in COGA^36^. Conventional Fourier transform methods^37^ were used to calculate coherence. Coherence measures were generated at the following frequencies: theta (3–7 Hz), alpha (7–12 Hz), beta (12–28 Hz).

Genotyping for the COGA EA participants was performed in batches using the Illumina 1 M, Illumina OmniExpress, Illumina 2.5 M, and Smokescreen arrays. Details have previously been reported^38^. Briefly, a pruned set of 47,000 variants that were genotyped on all platforms and had minor allele frequencies >10% in the combined samples, Hardy–Weinberg equilibrium (HWE) *p*-values > 0.001, missing rates <2%, and were not in linkage disequilibrium (LD; defined as *R*^2^ < 0.5) were used to assess reported pedigree structure using identity-by-descent calculations in PLINK^39^. Family structures were altered as needed and SNP genotypes were tested for Mendelian inconsistencies with the revised family structure^40^. Genotype inconsistencies were set to missing. Imputation was to 1000 Genomes (EUR and AFR, Phase 3, b37, October 2014; build hg19) using SHAPEIT2^41^ and then Minimac3^42^. Imputed SNPs with INFO scores <0.30 or individual genotype probability <0.90 were excluded, as were palindromic SNPs, monomorphic SNPs, SNPs with a genotyping rate of <95%, SNPs that were not in HWE, and SNPs with a minor allele frequency <0.05%. In total, 6,881,872 SNPs passed quality control and were available for analysis.

Polygenic risk scores (PRS) were constructed using the results published PGC GWAS of SCZ (36,989 cases and 113,075 controls)^32^, BiP (20,352 cases and 31,358 controls)^43^, and MDD (135,458 cases and 344,901 controls)^44^. To obtain independent sets of SNPs, we performed linkage disequilibrium (LD) based “clumping” in the COGA EA sample (founders only) using a 500 kb physical distance and an LD threshold of *r*^2^ ≥ 0.1. For each COGA participant, we derived an individual-level risk score by weighting the number of risk alleles at each independent SNP by the natural logarithm of its odds ratio (OR) and summing this quantity across independent SNPs. scores based on nine progressively more inclusive thresholds of statistical significance in each discovery GWAS: *p* < 0.0001, 0.001, 0.01, 0.05, 0.1, 0.2, 0.3, 0.4, and 0.5. To aid in the interpretation, PRS values were transformed to *Z*-scores so that effect sizes could be expressed as SD of coherence values per SD of PRS.

### Statistical analysis

We examined the trajectory of association among SCZ PRS with EEG coherence (theta, alpha, and beta). Association trajectories of PRS with EEG coherence were calculated as described in detail previously^22,28^. Given previous research demonstrating sex differences both in measures of EEG^28^, the clinical presentation of SCZ^29^, and polygenic effects on neuropsychiatric illnesses^22,30,31^, sex-specific models were examined. Covariates in the model included genotyping array, ancestral principal components (PCs 1–3), and a sex × PRS interaction term. Weights for individuals were adjusted to account for multiple observations on single individuals and co-presence of related individuals (e.g., siblings). Given the mixed evidence regarding the influence of substance use on the onset of psychosis^45^, and the potential for confounding introduced by the enrichment of substance use/disorders within the COGA derived sample, we examined models adjusted for the regular use of alcohol and nicotine, and ever use of cannabis on PRS-EEG coherence associations. We next examined specific genetic variants included in the PRS to determine whether any individual SCZ loci are driving this finding. Note that 101 of 108 loci reported by the PGC were available for analysis in the COGA sample.

We examined similar models independently including PRS for related conditions, BiP and MDD^43,44^, to determine the similarities and differences in developmental trajectories of EEG coherence for polygenic influences across all three conditions. Only the single most significant PRS were examined, selected on the basis of the EEG coherence association findings. All analyses were conducted in Matlab and were subject to an FDR correction for multiple testing; requiring *p* < 10^–4^ for significant associations based on an FDR less than 0.01.

## Results

**Fig. 2:**
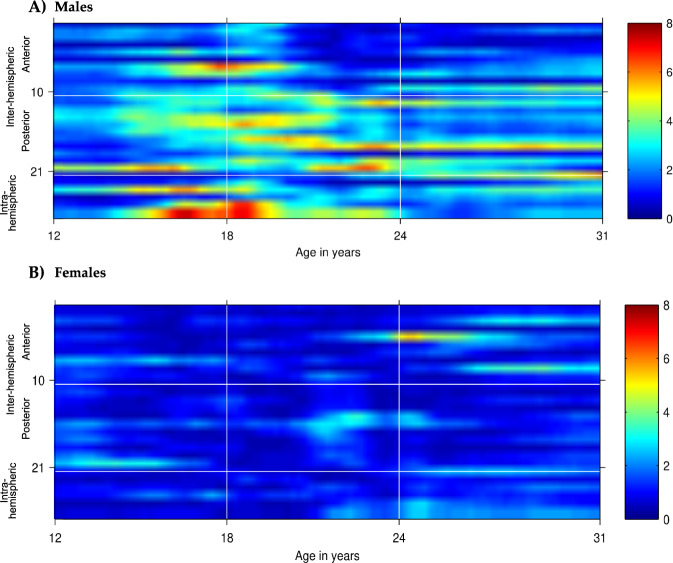
Association of SCZ PRS with interhemispheric and intrahemispheric high-alpha (9–12 Hz) EEG coherence networks in males (A) and females (B) from ages 12–31. Age is represented on the *x*-axis and coherences are represented on the *y*-axis, with numbering 1–27 corresponding to coherences between bipolar electrode pairs illustrated in Fig. 1. Regional divisions are marked with horizonal white grid lines: 1–10 anterior (frontal-central); 11–21 posterior (central-parietal-occipital); 22–27 left and right/anterior and posterior intrahemispheric. Color values in the main panels indicate levels of significance (*p*-values on a negative log 10 scale) as indicated by the color bars on the right.

We found that SCZ PRS were associated with *increased* EEG coherence in the theta and alpha frequencies in COGA participants. Findings for PRS based on the *p* < 0.05 *threshold* withstood a multiple test correction (FDR < 0.010), with the majority of coherence pairs in these frequency bands showing strong association with SCZ PRS (effect sizes ranged from 0.15 to 0.21 with all *p*-values < 10^–4^, Table 1; see also Fig. 2). We observed significant differences based on age and sex (Fig. 2 and Supplemental Figs. 1–6). Among males, the most robust associations were observed at ages 15–19 between PRS and high-alpha parietal-occipital (P4-O2 -- P3-O1), central-parietal (P7-P3 -- T7-C3, P8-P4 -- T8-C4, T7-P7 -- CZ-PZ, C3-P3 -- CZ-PZ), as well as right intrahemispheric (P8-P4--F8-F4, P8-P4--T8-C4) coherence pairs. Associations between PRS and high-alpha coherence were less robustly associated among females, with associations between PRS and fronto-central (F3-C3 -- F7-T7, FZ-CZ -- F8-T8) coherence pairs only observed at ages 24–26. Results are detailed in Table 1. In PRS-EEG coherence association models including alcohol, nicotine, and cannabis use frequency as covariates, results were largely unchanged (see Supplemental material).

**Table 1:** First quartile of association beta coefficients and *p*-values of SCZ PRS with high-alpha EEG coherence in males and females ages 12–31 with −log10 transformation.

		Ages 12–17		Ages 18–25		Ages 26–31	
		Male	Female	Male	Female	Male	Female
*Frontal-central* *sagittal*		−log10 *p*-value		−log10 *p*-value		−log10 *p*-value	
1	F8-T8--F7-T7	0.83	0.69	2.52	0.75	0.76	0.98
2	F4-C4--F3-C3	2.35	0.69	1.34	0.53	1.88	1.17
3	F3-C3--F8-T8	2.13	1.44	2.23	1.02	0.68	2.88
4	F4-C4--F7-T7	0.56	0.61	1.52	0.51	0.71	0.41
5	F3-C3--F7-T7	3.07	1.23	1.96	2.82	2.10	3.72
6	F4-C4--F8-T8	0.58	0.72	1.00	1.00	1.93	1.94
7	FZ-CZ--F7-T7	**4.77**	0.87	**4.43**	1.04	2.30	1.25
8	FZ-CZ--F3-C3	3.97	2.41	2.85	1.24	2.62	1.09
9	FZ-CZ--F8-T8	1.31	1.31	1.21	1.18	0.56	3.41
10	FZ-CZ--F4-C4	2.36	1.23	3.14	1.55	3.87	1.18
*Central-parietal sagittal*							
11	T8-P8--T7-P7	3.17	0.70	3.97	0.80	2.01	0.87
12	C4-P4--C3-P3	3.33	1.29	**4.50**	0.79	3.61	0.44
13	C3-P3--T8-P8	3.13	1.03	2.22	0.91	1.81	1.14
14	C4-P4--T7-P7	**4.14**	0.90	**4.62**	0.99	2.47	0.52
15	C3-P3--T7-P7	**4.47**	0.64	**4.77**	2.45	1.89	1.49
16	C4-P4--T8-P8	3.20	1.98	**2.77**	2.70	1.59	1.61
17	T7-P7--CZ-PZ	3.09	1.17	**5.15**	1.54	2.87	1.12
18	C3-P3--CZ-PZ	2.52	1.62	**5.18**	1.35	**4.97**	1.62
19	T8-P8--CZ-PZ	1.77	0.91	2.10	1.19	0.93	0.57
20	C4-P4--CZ-PZ	2.64	1.64	**4.12**	1.17	3.32	1.32
*Parietal-occipital sagittal*							
21	P4-O2--P3-O1	**5.24**	3.09	**5.38**	1.22	2.60	1.29
*Intrahemispheric lateral*							
22	T7-C3--F7-F3	0.66	0.84	2.48	0.99	**4.87**	2.50
23	P7-P3--F7-F3	1.56	0.56	1.64	0.94	1.87	0.89
24	P7-P3--T7-C3	**4.93**	0.97	2.99	1.31	3.55	1.83
25	T8-C4--F8-F4	1.14	1.86	2.73	1.31	2.37	1.19
26	P8-P4--F8-F4	**4.36**	0.44	3.64	1.55	1.79	1.68
27	P8-P4--T8-C4	**6.42**	0.76	**4.56**	2.01	2.43	2.16

All values in bold meet the false-discovery rate criterion of 10^−4^. Coherences between bipolar electrode pairs are numbered from 1–27 and organized by regions as illustrated in Fig. 1 and explained in its caption.

**Fig. 3:**
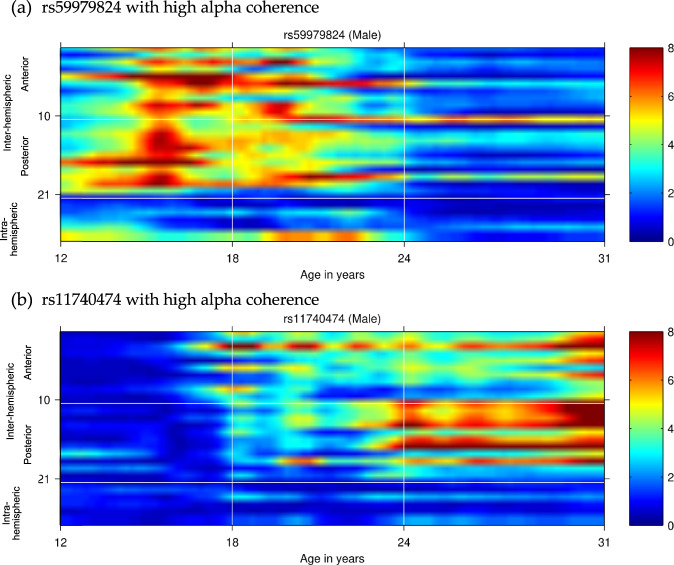
Associations of rs59979824 (top panel a) and rs11740474 (bottom panel b) with high-alpha EEG coherence in males from ages 12–31. Age is represented on the *x*-axis and coherences are represented on the *y*-axis, with numbering 1–27 corresponding to coherences between bipolar electrode pairs illustrated in Fig. 1. Regional divisions are marked with horizontal white grid lines: 1–10 anterior (frontal-central); 11–21 posterior (central-parietal-occipital); 22–27 left and right intrahemispheric. Color values in the main panels indicate levels of significance (*p*-values on a negative log 10 scale) as indicated by the color bars on the right.

Of the 101 genome-wide associated SNPs examined, male-only associations of alpha EEG coherence with rs59979824 (an intergenic variant on chromosome 2) and rs11740474 (*GALNT10* variant on chromosome 5) withstood a multiple test correction (Fig. 3, Supplemental Fig. 3, and Supplemental Table 1); Fig. 3 displays the association of rs59979824 (panel A) and rs11740474 (panel B) with high-alpha EEG coherence in males from ages 12–32. Associations of rs59979824 with high-alpha EEG coherence were observed across several fronto-central electrode pairs, with effects peaking between ages 14–24. In contrast, the majority of associations observed between rs11740474 and fronto-central high-alpha coherence occurred between ages 25–31.

**Fig. 4:**
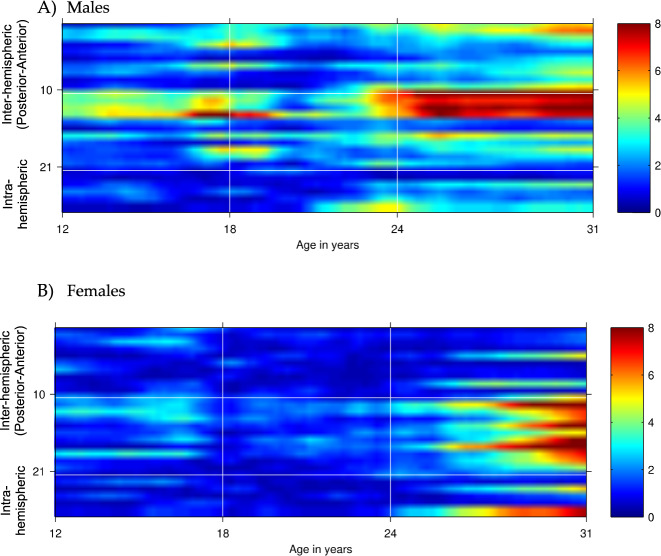
Association of depression PRS with interhemispheric and intrahemispheric high-alpha EEG coherence networks in males (A) and females (B) from ages 12–31. Age is represented on the *x*-axis and coherences are represented on the *y*-axis, with numbering 1–27 corresponding to coherences between bipolar electrode pairs illustrated in Fig. 1. Regional divisions are marked with horizontal white grid lines: 1–10 anterior (frontal-central); 11–21 posterior (central-parietal-occipital); 22–27 left and right intrahemispheric. Color values in the main panels indicate levels of significance (*p*-values on a negative log 10 scale) as indicated by the color bars on the right.

**Fig. 5:**
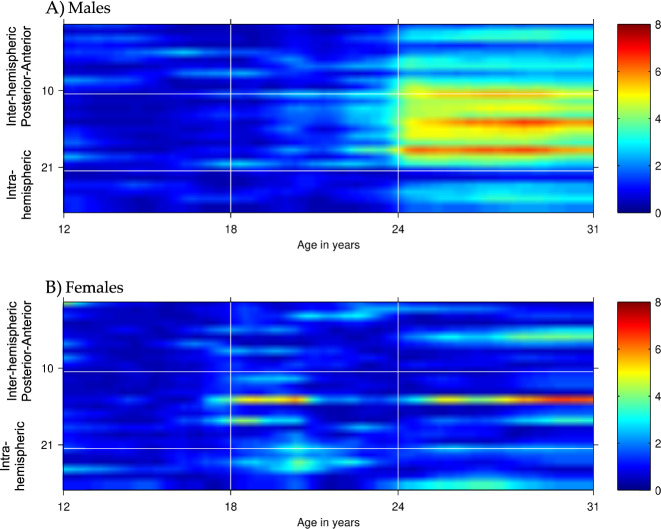
Association of bipolar disorder PRS with interhemispheric and intrahemispheric high-alpha EEG coherence networks in males (A) and females (B) from ages 12–31. Age is represented on the *x*-axis and coherences are represented on the *y*-axis, with numbering 1–27 corresponding to coherences between bipolar electrode pairs illustrated in Fig. 1. Regional divisions are marked with horizontal white grid lines: 1–10 anterior (frontal-central); 11–21 posterior (central-parietal-occipital); 22–27 left and right intrahemispheric. Color values in the main panels indicate levels of significance (*p*-values on a negative log 10 scale) as indicated by the color bars on the right.

Significant positive associations were observed among PRS for SCZ, BiP and MDD (SCZ and MDD PRS correlations ranged from 0.2 to 0.4, whereas SCZ and BiP PRS correlations were as high as 0.9). Both BiP and MDD PRS were also associated with EEG coherence. For MDD PRS, the most robust associations were observed with central-parietal high-alpha coherence in females ages 25 and older and in central-temporal high-alpha coherence in males ages 25 and older (Fig. 4 and Supplemental Table 2). For BiP PRS, the most robust associations were observed with central-frontal (FZ-CZ—F3-C3) high-alpha coherence in females ages 12–18 and central-parietal (P8-P4--T8-C4) high-alpha coherence in males 12–18 (Fig. 5 and Supplemental Table 3).

## Discussion

We report the robust associations of polygenic risk for SCZ with developmental trajectories of neural connectivity among unaffected individuals: in particular, we observed that males with higher polygenic risk for SCZ exhibit increased EEG coherence in the alpha frequency band in posterior brain regions between the ages of 15 and 19. Although the “disconnection hypothesis”^18^ has been one of the main etiological models of SCZ for decades and has been generally supported^46^, empirical evidence has been somewhat mixed, pointing to more complex abnormalities in the connectivity pattern including both increases and decreases of connectivity depending on specific location, subjects’ state (e.g., during a task vs. in the resting-state), and neuroimaging modality^47–49^. Findings from our study of increased connectivity correspond well to previous work demonstrating higher resting-state alpha EEG coherence both within and across hemispheres among individuals with SCZ and in their relatives^5^. While *increased* connectivity observed among individuals with higher SCZ PRS may seem counterintuitive to many dysconnectivity hypotheses in psychotic illness, several previous studies have found similar patterns of increased connectivity among those with SCZ and their relatives^5,50–54^. For example, Kam et al.^5^ found that individuals with SCZ exhibited greater connectivity, and emphasized that, as in our study, EEG was recorded during rest as opposed to during a cognitive task. They suggest that this pattern of increased neural synchronization may reflect network activity in the resting-state that is less selectively organized among individuals at risk for SCZ, consistent with suggestions that SCZ may be associated with inefficient neural processing^55–57^.

Further, we found striking sex differences wherein polygenic risk for SCZ was associated with increased parietal-occipital and -central alpha coherence among males ages 15–19, and with fronto-central alpha coherence among females ages 25–31, mapping onto key periods of risk for the onset of psychotic illness in each group. Interestingly, this was in contrast to findings for polygenic risk for MDD, where the most robust associations were observed with central-parietal alpha coherence in both males and females at age 25, also mirroring the later clinical onset of MDD. Findings from this study support a century of theory positing SCZ as a neurodevelopmental disorder, with perturbations in brain development occurring well before the onset of symptoms. We note however, that these PRS effects are subtle (*R*^2^ values < 0.02). Findings also demonstrate the critical importance of examining sex differences in neuropsychiatric PRS-EEG coherence associations throughout development.

We observed important sex and developmental effects in the association of two individual genome-wide significant SCZ risk loci with high-alpha coherence; effects of intergenic chromosome 2 variant rs59979824 were observed among males 14–24, whereas the effect of *GALNT10* variant rs11740474 on chromosome 5 was most robustly observed after age 25. rs59979824 has been associated with SCZ in three independent studies^32,58,59^ and a fourth study that examined overlapping SCZ and autism spectrum disorder (ASD) GWAS variants^60^. There is some evidence, albeit weak, from the Braineac database (http://www.braineac.org/) that rs59979824 is associated with the expression of *TMEFF2*, which has also been previously associated with SCZ^61,62^. Recent investigations have suggested that *TMEFF2* may modulate the level of sarcosine (N-methylglycine) and therefore the activity of the glycine transporter type I (GlyT1)^61,62^. One of the two primary pharmacological possibilities for enhancing function of the NMDA receptor relies on inhibiting the glycine transporter type I (GlyT-1) with sarcosine, bitopertin (RG1678), or ALX-5407^63^. Sarcosine has shown promising early results in ameliorating cognitive and negative symptoms in SCZ. *GALNT10* variant rs11740474 has also been implicated in several previous studies of SCZ^32,58,59,64^ and one study of ASD^60^.

Recent studies have shown that polygenic risk for SCZ influences working memory in both individuals with^23^ and without SCZ^24^, with trend level evidence of the relation of SCZ PRS with functional connectivity of fronto-parietal network supporting numerical working memory in healthy young adults^24^. Poorer performance and slower processing speed on various cognitive tasks have also been observed among individuals with SCZ^65^. A recent study found that microstructural abnormalities in the callosal white matter fibers connecting bilateral temporal lobe cortices contribute to poor neuropsychological performance and severe negative symptom in patients with schizophrenia^66^. Taken together, there is strong evidence that these cognitive deficits are involved in risk for SCZ. Fortunately, promising clinical trials of working memory and processing speed training for individuals with SCZ are underway^67^. However, further research is needed to understand if genetic risk influences early neural developmental factors to give rise to cognitive deficits, that may increase risk for SCZ.

SCZ, BiP and MDD are genetically correlated (rg = 0.34–0.70) suggesting pleiotropic effects of variants across these three disorders. Despite the genetic correlations consistently observed among these conditions, patterns of associations with SCZ PRS differed markedly from those observed for BiP and MDD PRS (Supplemental Fig. 4). Whereas influences of SCZ PRS were observed only for males aged 16–20 (Fig. 2), both BiP PRS and MDD PRS had influences in both males and females, with BiP effects appearing later in females, and MDD effects manifesting in females after age 24 (Figs. 4–5). Additionally, SCZ PRS effects are more widespread throughout the brain, impacting frontal, central, posterior, and both interhemispheric and intrahemispheric electrode pairs, whereas effects of BiP and MDD PRS were largely interhemispheric and central-posterior. While some of these observed effects are shared across all three conditions^25^, the similarities and distinctions are in line with what is known regarding the shared and unique genetic and neural contributions to SCZ, BiP, and MDD.

The current study uncovered neurodevelopmental patterns of brain connectivity in adolescents and young adults who did not have a clinical diagnosis of SCZ (or BiP, MDD) but were at varying polygenic liability to SCZ. We also demonstrated important sex and developmental differences in these brain connectivity patterns. This is the first study to our knowledge that has linked neuropsychiatric PRS to developmental trajectories of brain connectivity. The most notable strength of this study includes the large genetic sample of males and females assessed with repeated measures of connectivity throughout adolescence and young adulthood—a key neurodevelopmental period. However, we note that the power to detect significant associations with EEG coherence trajectories was impacted by the discovery GWAS sample sizes, and the disorder-specific genetic architecture; that is, SCZ and BiP have greater heritability than MDD and relatedly the discovery sample sizes for SCZ GWAS are greater than sample sizes for BiP and MDD. In addition, an important limitation of the current study is that only EA individuals were included due to the majority EA composition of these discovery GWAS, as cross-ancestry predictions have the potential to yield biased (or confounded) estimates^68^. Future directions of this work include cross-ancestry replication of these effects using new results from the Genomic Psychiatry Cohort (GPC)^69^ and joint analyses of other SCZ cohorts with available clinical data, especially as related to age at onset and specific SCZ symptom clusters. Findings from this study of unaffected individuals from the general population (i.e., in the absence of clinical features of SCZ or a family history of SCZ) should be compared with patterns observed in clinical samples of those with SCZ, BiP, and MDD. In addition, EEG studies could be paired with fMRI to combine the advantages of the superior spatial resolution of MRI and the superior temporal resolution of EEG, to better understand findings from both modes of measuring functional connectivity.

## Supplementary information

Supplemental Figures and Tables
