# Supplementary material for: The association of polygenic risk for schizophrenia, bipolar disorder, and depression with neural connectivity in adolescents and young adults: examining developmental and sex differences
Source: Transl Psychiatry. 2021 Jan 14;11:54. doi: 10.1038/s41398-020-01185-7 (PMC7809462; doi:10.1038/s41398-020-01185-7)
Supplement: Supplementary file 1 — Supplemental Figures and Tables [file 41398_2020_1185_MOESM1_ESM.docx]

**Supplemental Figure 1.** Association of Schizophrenia PRS (9 levels) with EEG coherence in 7 frequency bands (low theta, high theta, low alpha, high alpha, low beta, mid beta, high beta) in males, presented in three panels representing separate age ranges: 12-18 (Top Panel), 18-25 (middle panel), 25-31 (bottom panel). Coherences are represented on the Y-axis, with numbering 1-27 corresponding to coherences between bipolar electrode pairs illustrated in Figure 1. Regional divisions are marked with horizontal white grid lines: 1-10 Anterior (Frontal-Central); 11-21 Posterior (Central-Parietal-Occipital); 22-27 Left and Right intrahemispheric. Successive frequency bands are shown along the x-axis; within each frequency band the 9 levels are shown in ascending order. For each age range, the color of the box at each combination of frequency band, PRS level, and coherence represents the significance (-log_10 p-value) of the 1st quartile of the 32 association calculations with age center in the age range, as indicated by the color bars on the right.

Inter-hemispheric

Posterior-Anterior

Intra-

hemispheric

Inter-hemispheric

Posterior-Anterior

Intra-

hemispheric

Inter-hemispheric

Posterior-Anterior

Intra-

hemispheric

Low theta High theta Low alpha High alpha Low beta Mid beta High beta

Low theta High theta Low alpha High alpha Low beta Mid beta High beta

Low theta High theta Low alpha High alpha Low beta Mid beta High beta

**Supplemental Figure 2.** Association of Schizophrenia PRS (9 levels) with EEG coherence in 7 frequency bands (low theta, high theta, low alpha, high alpha, low beta, mid beta, high beta) in females, presented in three panels representing separate age ranges: 12-18 (Top Panel), 18-25 (middle panel), 25-31 (bottom panel). Coherences are represented on the Y-axis, with numbering 1-27 corresponding to coherences between bipolar electrode pairs illustrated in Figure 1. Regional divisions are marked with horizontal white grid lines: 1-10 Anterior (Frontal-Central); 11-21 Posterior (Central-Parietal-Occipital); 22-27 Left and Right intrahemispheric. Successive frequency bands are shown along the x-axis; within each frequency band the levels are shown in ascending order. For each age range, the color of the box at each combination of frequency band, PRS level, and coherence represents the significance (-log_10 p-value) of the 1st quartile of the 32 association calculations with age center in the age range, as indicated by the color bars on the right.

Intra-

hemispheric

Inter-hemispheric

Posterior-Anterior

Intra-

hemispheric

Inter-hemispheric

Posterior-Anterior

Intra-

hemispheric

Inter-hemispheric

Posterior-Anterior

Low theta High theta Low alpha High alpha Low beta Mid beta High beta

Low theta High theta Low alpha High alpha Low beta Mid beta High beta

Low theta High theta Low alpha High alpha Low beta Mid beta High beta

**Supplemental Figure 3.** Association of 101 SNPs contributing to the SCZ PRS with high alpha EEG coherence networks among males ages 12-18 (top panel), 18-25 (middle panel), and 25-31 (bottom panel). Individual SNPs and corresponding chromosome are represented on the X-axis and coherences are represented on the Y-axis, with numbering 1-27 corresponding to coherences between bipolar electrode pairs illustrated in Figure 1. Regional divisions are marked with horizontal white grid lines: 1-10 Anterior (Frontal-Central); 11-21 Posterior (Central-Parietal-Occipital); 22-27 Left and Right intrahemispheric.

For each age range, the color of the box at each combination of SNP and coherence pair represents the significance (-log_10 p-value) of the 1st quartile of the 32 association calculations with age center in the age range, as indicated by the color bars on the right.

nh

Intra-

hemispheric

Inter-hemispheric

Posterior-Anterior

Intra-

hemispheric

Inter-hemispheric

Posterior-Anterior

Intra-

hemispheric

Inter-hemispheric

Posterior-Anterior

**Supplemental Figure 4.** Schematic of comparison of PRS effects on high alpha EEG coherence across three neuropsychiatric conditions. Red lines represent connections that showed most statistically significant correlation with the PRS, while subthreshold findings are represented in yellow. See Fig. 1 for detailed explanation of scalp topography and electrode labeling of coherences.

Schizophrenia

(males, 16-20)

Major Depression (both, 24+)

Bipolar Disorder

(females, 17+; males, 24)+

**Supplemental Figure 5.** Panel A displays a quantile-quantile plot (QQ plot) observed p-values from the association analysis of high alpha coherence with SCZ PRS among males. Panel B displays the distribution of the 2,592 (27 coherence pairs x 96 age centers) observed p-values from the association analysis of high alpha coherence with SCZ PRS values for males. The blue line represents an estimate of the distribution of the p-values of the cases in which there were no genotypic associations in the data on the conservative assumption that there were 2000 such cases. This plot corresponds to Fig. 1 in Storey and Tibshirani [76].

**Supplemental Figure 6.** Distribution of p-values less than .1 in which each interval on the x axis (.005) is half the width as that in Supplemental Figure 5B in order to show the evaluation of the most significant p-values. The cyan line represents conservative estimate of the distribution of the p-values of the cases in which there were no genotypic associations in the data. The red line marks the boundary between, on the left, the p-values considered significant in the analysis and, on the right, those not. The area of the box bounded by the cyan and red lines touching the origin represents the false discoveries at a rate somewhat less than 5%.

**Supplemental Table 1.** First Quartile of Association beta coefficients and p-values of 101 SNPs (p-threshold <0.05) with low high alpha EEG coherence in males and females ages 12-31 with -log10 transformation. All values in bold meet the false discovery rate criterion of 10^^-4^; **10^^-6^.

| *Chr* | *SNP* | *BP Location* | *A1;A2* | *Age Range Specific log10 (p-value)* | | |
| --- | --- | --- | --- | --- | --- | --- |
|  |  |  |  | *12-18* | *18-25* | *25-31* |
| 2 | rs59979824 | 193,848,340 | C;A | **6.74**** | **5.94** | 2.82 |
| 3 | rs832187 | 63,833,050 | C;T | 1.50 | 1.66 | **4.05** |
| 3 |  | 180,594,593 | T;A | **4.78** | 2.26 | 1.50 |
| 5 | rs4388249 | 109,036,066 | C:T | 1.13 | **4.77** | 2.71 |
| 5 | rs3849046 | 137,851,192 | C;T | 1.94 | 1.95 | **5.02** |
| 5 | rs111294930 | 152,177,121 | A:G | 1.85 | 3.07 | **4.59** |
| 5 | rs11740474 | 153,680,747 | A;T | 2.44 | **5.27** | **7.71**** |
| 6 |  | 73,155,701 | C | 2.01 | **4.04** | **8.48**** |
| 6 |  | 84,280,274 | G | 2.71 | **4.38** | **4.31** |
| 6 | rs117074560 | 96,459,651 | C;T | **4.01** | 2.20 | 2.53 |
| 8 | rs10503253 | 4,180,844 | C;A | **4.23** | 1.94 | 1.48 |
| 8 | rs4129585 | 143,312,933 | A;C | 2.36 | 1.70 | **4.25** |
| 11 |  | 46,350,213 | AG | 2.01 | **4.06** | 3.56 |
| 11 | rs2514218 | 113,392,994 | C;T | 1.70 | 2.53 | **4.24** |
| 11 | rs77502336 | 123,394,636 | G;C | 1.27 | 1.85 | **4.15** |
| 12 | rs679087 | 29,917,265 | C;A | 1.34 | 1.92 | **4.10** |
| 12 | rs10860964 | 103,596,455 | T;C | 3.32 | **4.42** | 3.02 |
| 15 | rs12903146 | 61,854,663 | G;A | **5.12** | 2.12 | 2.20 |
| 15 | rs8042374 | 78,908,032 | A;G | **4.20** | **4.54** | 2.92 |
| 16 | rs12691307 | 29,939,877 | A | **4.64** | 3.76 | 2.11 |

**Supplemental Table 2.** First Quartile of Association beta coefficients and p-values of Bipolar Disorder PRS (p-threshold <0.05) with low theta and high alpha EEG coherence in males and females ages 12-31 with -log10 transformation. All values in bold meet the false discovery rate criterion of 10^^-4^**. C**oherences between bipolar electrode pairs are numbered from 1-27 and organized by regions as illustrated in Figure 1 and explained in its caption.

|  |  | **Ages 12-17** | | **Ages 18-25** | | **Ages 26-31** | |
| --- | --- | --- | --- | --- | --- | --- | --- |
|  |  | *male* | *female* | *male* | *female* | *male* | *female* |
| *Frontal central sagittal* | | -log10 p-value | | -log10 p-value | | -log10 p-value | |
| 1 | F8-T8--F7-T7 | 1.42 | 1.27 | 1.09 | 1.18 | 1.58 | 0.58 |
| 2 | F4-C4--F3-C3 | 0.51 | 0.75 | 0.99 | 2.20 | 3.16 | 1.81 |
| 3 | F3-C3--F8-T8 | 1.60 | 0.82 | 1.61 | 2.61 | 3.33 | 1.36 |
| 4 | F4-C4--F7-T7 | 0.94 | 0.49 | 1.01 | 1.00 | 1.54 | 0.48 |
| 5 | F3-C3--F7-T7 | 2.01 | 1.22 | 1.49 | 1.63 | 2.15 | 2.67 |
| 6 | F4-C4--F8-T8 | 1.04 | 0.49 | 1.98 | 0.62 | 2.85 | 3.73 |
| 7 | FZ-CZ--F7-T7 | 0.74 | 1.66 | 2.28 | 0.67 | 3.19 | 1.98 |
| 8 | FZ-CZ--F3-C3 | 1.97 | 0.71 | 1.77 | 1.93 | 2.21 | 0.56 |
| 9 | FZ-CZ--F8-T8 | 1.61 | 1.03 | 2.13 | 0.97 | 3.02 | 1.14 |
| 10 | FZ-CZ--F4-C4 | 1.03 | 0.85 | 1.75 | 1.11 | **4.03** | 0.82 |
| *Central-Parietal sagittal* | | |  |  |  |  |  |
| 11 | T8-P8--T7-P7 | 1.05 | 0.62 | 2.97 | 1.04 | **5.46** | 1.18 |
| 12 | C4-P4--C3-P3 | 0.45 | 0.77 | 1.98 | 2.39 | **4.33** | 1.41 |
| 13 | C3-P3--T8-P8 | 0.69 | 0.70 | 2.77 | 1.10 | **4.87** | 1.55 |
| 14 | C4-P4--T7-P7 | 1.01 | 0.82 | 2.59 | 1.03 | **4.14** | 0.48 |
| 15 | C3-P3--T7-P7 | 0.66 | 1.38 | 2.66 | **4.83** | **6.21** | **6.25** |
| 16 | C4-P4--T8-P8 | 0.67 | 1.17 | 2.06 | 0.62 | **5.18** | 1.19 |
| 17 | T7-P7--CZ-PZ | 0.89 | 1.11 | 2.53 | 0.91 | **4.70** | 1.71 |
| 18 | C3-P3--CZ-PZ | 0.94 | 1.53 | 1.71 | 2.70 | **4.04** | 3.17 |
| 19 | T8-P8--CZ-PZ | 0.83 | 1.18 | 3.52 | 1.74 | **6.30** | 0.93 |
| 20 | C4-P4--CZ-PZ | 1.41 | 0.65 | 1.63 | 1.16 | **4.40** | 1.05 |
| *Parietal-Occipital sagittal* | | |  |  |  |  |  |
| 21 | P4-O2--P3-O1 | 1.61 | 0.98 | 2.57 | 1.55 | 3.42 | 1.06 |
| *Intrahemispheric lateral* | | |  |  |  |  |  |
| 22 | T7-C3--F7-F3 | 0.74 | 1.00 | 0.74 | 2.50 | 1.62 | 2.31 |
| 23 | P7-P3--F7-F3 | 0.40 | 1.05 | 0.84 | 1.90 | 0.80 | 1.64 |
| 24 | P7-P3--T7-C3 | 1.21 | 1.26 | 1.07 | 2.95 | 2.47 | 1.59 |
| 25 | T8-C4--F8-F4 | 0.89 | 1.91 | 1.38 | 2.43 | 2.64 | 0.95 |
| 26 | P8-P4--F8-F4 | 1.27 | 0.53 | 1.23 | 1.15 | 3.20 | 1.74 |
| 27 | P8-P4--T8-C4 | 0.74 | 1.00 | 0.74 | 2.50 | 1.62 | 2.31 |

**Supplemental Table 3.** First Quartile of Association beta coefficients and p-values of MDD PRS (p-threshold <0.05) with low theta and high alpha EEG coherence in males and females ages 12-31 with -log10 transformation. All values in bold meet the false discovery rate criterion of 10^^-4^**. C**oherences between bipolar electrode pairs are numbered from 1-27 and organized by regions as illustrated in Figure 1 and explained in its caption.

|  |  | **Ages 12-17** | | **Ages 18-25** | | **Ages 26-31** | |
| --- | --- | --- | --- | --- | --- | --- | --- |
|  |  | *male* | *female* | *male* | *female* | *male* | *female* |
| Coherence *Frontal central sagittal* | | -log10 p-value | | -log10 p-value | | -log10 p-value | |
| 1 | F8-T8--F7-T7 | 1.792 | 2.031 | 3.815 | 2.031 | **5.05** | 0.553 |
| 2 | F4-C4--F3-C3 | 3.067 | 1.588 | 3.245 | 1.588 | **5.903** | 1.27 |
| 3 | F3-C3--F8-T8 | 3.266 | 2.67 | 2.986 | 2.67 | 3.837 | 1.343 |
| 4 | F4-C4--F7-T7 | 3.757 | 1.511 | 2.891 | 1.511 | 2.749 | 0.624 |
| 5 | F3-C3--F7-T7 | 1.676 | 0.689 | 1.492 | 0.689 | 1.918 | 3.899 |
| 6 | F4-C4--F8-T8 | 0.989 | 1.365 | 0.995 | 1.365 | 2.915 | 0.388 |
| 7 | FZ-CZ--F7-T7 | 3.767 | 1.297 | 3.211 | 1.297 | 3.627 | 0.894 |
| 8 | FZ-CZ--F3-C3 | 2.146 | 0.615 | 2.101 | 0.615 | **4.113** | 0.977 |
| 9 | FZ-CZ--F8-T8 | 0.94 | 1.266 | 1.514 | 1.266 | 3.078 | 3.852 |
| 10 | FZ-CZ--F4-C4 | 1.37 | 1.484 | 3.625 | 1.484 | **4.801** | 0.676 |
| *Central-Parietal sagittal* | | |  |  |  |  |  |
| 11 | T8-P8--T7-P7 | **4.231** | 2.324 | **5.63** | 2.324 | **9.479** | **4.13** |
| 12 | C4-P4--C3-P3 | **4.311** | 2.431 | **5.628** | 2.431 | **7.193** | **8.805** |
| 13 | C3-P3--T8-P8 | **4.745** | 3.152 | **4.77** | 3.152 | **8.434** | **5.472** |
| 14 | C4-P4--T7-P7 | **7.136** | 2.677 | **5.663** | 2.677 | 7.289 | **4.673** |
| 15 | C3-P3--T7-P7 | 2.33 | 1.512 | 1.857 | 1.512 | 1.968 | **7.017** |
| 16 | C4-P4--T8-P8 | 1.072 | 1.466 | 1.84 | 1.466 | 1.14 | **4.271** |
| 17 | T7-P7--CZ-PZ | 3.393 | 2.158 | 3.911 | 2.158 | **4.844** | **6.534** |
| 18 | C3-P3--CZ-PZ | 1.455 | 0.543 | 3.156 | 0.543 | 2.911 | **8.513** |
| 19 | T8-P8--CZ-PZ | 3.211 | 3.212 | 2.866 | 3.212 | **4.245** | **5.645** |
| 20 | C4-P4--CZ-PZ | 2.445 | 1.027 | 2.458 | 1.027 | 2.812 | **5.066** |
| *Parietal-Occipital sagittal* | | |  |  |  |  |  |
| 21 | P4-O2--P3-O1 | 1.655 | 1.034 | 3.068 | 1.034 | 2.391 | 3.35 |
| *Intrahemispheric lateral* | | |  |  |  |  |  |
| 22 | T7-C3--F7-F3 | 0.953 | 0.614 | 1.789 | 0.614 | 1.03 | 1.898 |
| 23 | P7-P3--F7-F3 | 0.641 | 1.82 | 0.72 | 1.82 | 0.506 | 0.763 |
| 24 | P7-P3--T7-C3 | 1.407 | 0.548 | 2.286 | 0.548 | 3.686 | **4.028** |
| 25 | T8-C4--F8-F4 | 1.969 | 1.469 | 1.8 | 1.469 | 1.992 | 1.476 |
| 26 | P8-P4--F8-F4 | 1.439 | 1.157 | 2.423 | 1.157 | 1.964 | 0.892 |
| 27 | P8-P4--T8-C4 | 0.644 | 1.141 | **4.218** | 1.141 | 3.148 | 1.898 |
